# Recurrence rates in ocular non-infectious uveitis according to US FDA criteria or Rest of World criteria

**DOI:** 10.1186/s12348-022-00307-0

**Published:** 2022-09-14

**Authors:** Glenn J. Jaffe, Carlos E. Pavesio

**Affiliations:** 1 Department of Ophthalmology, Duke University, Durham, North Carolina USA; 2 Moorfields Eye Hospital, London, UK

Dear Editor.

PSV-FAI-001 was a phase 3, prospective, double-masked, multicenter study that began in August 2013 and was completed in March 2018 (clinicaltrials.gov, NCT01694186). The study was designed to examine efficacy and safety outcomes of a 0.2 μg/day fluocinolone acetonide insert (FAi) over 36-months in patients with non-infectious uveitis affecting the posterior segment of the eye [1].

The primary efficacy outcome defined at month 6 and was the proportion of eyes that had a uveitis recurrence. Secondary endpoints included the time to the first recurrence and the number of recurrences at 12 and 36 months. Recurrence was defined as a ≥ 2 step increase in number of anterior chamber cells per high powered field or ≥ 2 step increase in vitreous haze or a loss in best-corrected visual acuity of ≥15 letters or the need for supplemental therapy that included systemic, injectable and/or topical corticosteroids and/or systemic immunosuppressants).

Since the study was completed, the insert has been licensed for medical use in the USA for the treatment of chronic non-infectious uveitis affecting the posterior segment of the eye [2] and in Europe and the Middle East to prevent relapse in recurrent non-infectious uveitis affecting the posterior segment of the eye [3].

However, while PSV-FAI-001 was used to support the registration of YUTIQ® (branded name in the USA) and ILUVIEN® (branded name in Europe and the Middle East), the interpretation of the study outcomes differed between the health authorities appraising the applications. In Europe and the Middle East, the recurrence criteria detailed above were used.

In the USA, however, at the Type C meeting on March 10, 2015, the Division of Transplant and Ophthalmology Products did not agree to include the first criterion, anterior chamber cells, as they considered anterior and posterior uveitis to be two separate indications [4]. To address this issue, EyePoint Pharmaceuticals Inc. prepared statistical analysis plans, one for the USA submission and one for the Rest of the World (ROW) rather than to revise the definition in the study protocol. The USA registration was therefore based on the exclusion of ‘an increase of ≥ 2 steps in the number of cells in the anterior chamber’ and the primary endpoint was defined at Month 6. In contrast, the ROW registration includes the number of cells in the anterior chamber and the study data over a 36-month period. These differences are reflected in the prescribing information, with ILUVIEN treatment being defined over 36 months and YUTIQ over 6 (primary endpoint) and recurrence was also assessed at Month 12.

The differences in YUTIQ and ILUVIEN labelling and prescribing information may be confusing for readers, prescribers, and patients. Here we summarize, over 36 months, the outcomes (recurrence, time to first recurrence and number of recurrences) based on the measurements of recurrence defined in the USA and the Rest of the World (Table 1).

**Table 1:** Recurrence related outcomes over 36 months based on USA and the Rest of World measurements of recurrence

	Rest of World^**a**^		USA^**b**^	
	FAi (***N*** = 87)	Sham treated (***N*** = 42)	FAi (***N*** = 87)	Sham treated (***N*** = 42)
**Recurrence**				
No recurrence, %	34.5	2.4	43.7	7.1
Recurrence rate, %	65.5	97.6	56.3	92.9
*Observed, %*	*5.7*	*28.6*	8.0	21.4
*Imputed, %*	*59.8*	*69.0*	48.3	71.4
**Reason for imputation**				
Systemic treatments, %	28.7	11.9	31.0	21.4
Local injections, %	8.0	38.1	9.2	42.9
Topical medication, %	18.4	19.0	Not evaluated	
**Number of recurrences**				
Number of recurrences per eye, mean ± standard deviation	1.7 ± 2.4	5.3 ± 3.8	1.2 ± 2.0	4.0 ± 3.3
Eyes with ≤1 recurrence, %	67.8	14.3	78.2	26.1
Eyes with > 5 recurrences, %	8.0	40.5	2.3	26.2
**Time to first recurrence**				
Time to, days, median	657	70.5	1051	95

^a^Recurrence was defined as a ≥ 2 step increase in number of cells in the anterior chamber per high powered field or ≥ 2 step increase in vitreous haze or a loss in best-corrected visual acuity of ≥15 letters or the need for supplemental therapy (i.e., corticosteroids (systemic, injectable, or topical) or systemic immunosuppressants)

^b^Same recurrence criteria with the exclusion of ≥2 step increase in number of cells in the anterior chamber per high powered field and the use of topical corticosteroids to define a recurrence

The following relative differences were observed in the USA defined recurrence group versus the Rest of World defined group over a three-year period:

1. A longer median time to first recurrence in FAi- (1051 days vs. 657 days) and sham-treated eyes (95.0 days vs. 70.5 days).
2. Overall, there were a greater proportion of eyes that were free from recurrence in both FAi- and sham-treated eyes (Table 1).
3. Fewer mean number of recurrences for FAi- (1.2 vs. 1.7; USA vs. ROW) and sham-treated eyes (4.0 vs. 5.3).
4. A greater proportion of eyes with ≤1 recurrence in FAi- (78.2% vs. 67.8%) and sham-treated eyes (26.1% vs. 14.2%).
5. A smaller proportion of eyes with > 5 recurrences in FAi- (2.3% vs. 8.0%) and sham-treated eyes (26.2% vs. 40.5%).

These differences between FAi-treated and sham -treated eyes observed with both the US and ROW recurrence definitions suggest that non-infectious uveitis affecting the posterior segment of the eye can be managed effectively with a single FAi. Key FAi benefits to the patient include significantly delayed inflammation recurrence, and reduction in the recurrence frequency. These findings have implications for the management of patients in a clinical setting as they suggest that the recurrence of uveitis in the posterior segment of the eye can be well controlled using localized, long-term FAi-therapy.

Of note, when the ROW recurrence definition was applied there was a shorter time to inflammation recurrence, and more frequent recurrences when compared to that with the US definition. These differences can be explained by the US definition that did not account for recurrences related to anterior chamber inflammation. These data highlight the point that clinicians must be aware that topical anti-inflammatory therapy may be needed to manage recurrences, even when the posterior segment inflammation is still controlled. (see Table 1) The frequency and severity of these anterior chamber inflammation recurrences may dictate whether the clinician elects to manage the anterior chamber recurrences solely with topical therapy, or by placing an additional FAi .

## Data Availability

Not applicable. Please see References that support the current work.
